# LncRNA *DUXAP8* as a prognostic biomarker for various cancers: A meta-analysis and bioinformatics analysis

**DOI:** 10.3389/fgene.2022.907774

**Published:** 2022-08-15

**Authors:** Yongfeng Wang, Xianglai Jiang, Dongzhi Zhang, Yuanbin Zhao, Xiaoyong Han, Lihui Zhu, Jingyao Ren, Yubin Liu, Jiarong You, Haolan Wang, Hui Cai

**Affiliations:** ^1^ Graduate School, Ning Xia Medical University, Yinchuan, China; ^2^ General Surgery Clinical Medical Center, Gansu Provincial Hospital, Lanzhou, China; ^3^ Key Laboratory of Molecular Diagnostics and Precision Medicine for Surgical Oncology in Gansu Province, Gansu Provincial Hospital, Gansu, China; ^4^ NHC Key Laboratory of Diagnosis and Therapy of Gastrointestinal Tumor, Gansu Provincial Hospital, Lanzhou, China; ^5^ The First Clinical Medical College of Lanzhou University, Lanzhou, China

**Keywords:** long noncoding RNA, *DUXAP8*, prognosis, cancers, bioinformatics analysis

## Abstract

**Background:** Dual homeoboxes A pseudogene 8 (*DUXAP8*) is a newly discovered long noncoding RNA that has been shown to function as an oncogene in a variety of human malignant cancers. By integrating available data, this meta-analysis sought to determine the relationship between clinical prognosis and *DUXAP8* expression levels in diverse malignancies.

**Materials and methods:** A systematic search was performed to identify eligible studies from several electronic databases from their inception to 25 October 2021. Pooled odds ratios and hazard ratios with 95% CI were used to estimate the association between *DUXAP8* expression and survival. For survival analysis, the Kaplan-Meier method and COX analysis were used. Furthermore, we utilized Spearman’s correlation analysis to explore the correlation between *DUXAP8* and tumor mutational burden (TMB), microsatellite instability (MSI), the related genes of mismatch repair (MMR), DNA methyltransferases (DNMTs), and immune checkpoint biomarkers.

**Results:** Our findings indicated that overexpression of *DUXAP8* was related to poor overall survival (OS) (HR = 1.63, 95% CI, 1.49–1.77, *p* < 0.001). In addition, elevated *DUXAP8* expression was closely related to poor OS in several cancers in the TCGA database. Moreover, *DUXAP8* expression has been associated with TMB, MSI, and MMR in a variety of malignancies.

**Conclusion:** This study revealed that *DUXAP8* might serve as a prognostic biomarker and potential therapeutic target for cancer. It can be used to improve cancer diagnosis, discover potential treatment targets, and improve prognosis.

## Introduction

Cancer-related deaths have risen dramatically in recent years. Progress in anticancer drug delivery has resulted in tremendous improvements in cancer treatment outcomes, yet cancer survivors’ quality of life and prognosis remains dismal (Bahrami et al., 2018). In part, this is due to the lack of reliable biomarkers for the early identification of the majority of malignancies. In recent years, molecular biomarkers for multiple carcinomas have become more prevalent and may provide further clues for following the disease’s progression (Ilie et al., 2018). Consequently, it is imperative to seek new cancer markers to better characterize the clinical stage, metastasis, and prognosis of most malignancies at an earlier and more accurate point in time (Wang et al., 2021a, 2021b).

An RNA molecule that is more than 200 nucleotides in length but lacks an open reading frame is referred to as Long non-coding RNA (lncRNA) (Johnsson et al., 2014). It has emerged that lncRNAs have a role in a wide range of physiological and pathological processes. Epigenetic regulation, transcriptional and posttranscriptional regulation are only a few of the roles of lncRNAs in diseases (Fan et al., 2017). LncRNAs may play a critical role in the progression of cancer, as evidenced by recent research (Wang et al., 2021a). Collectively, lncRNAs serve as promising markers for cancer patients (Ma et al., 2017a).

Double homeobox A pseudogene 8 (*DUXAP8*) is a recently discovered lncRNA on 22q11.1. *DUXAP8* has a length of around 2,307 bp. *DUXAP8* is significantly overexpressed in cancer tissues compared to nearby non-tumor tissues, according to observations (Ma et al., 2017b; Du et al., 2019; Chen et al., 2020a; Chen et al., 2020b; He et al., 2020; Yin et al., 2020; Chen et al., 2021a). *DUXAP8* exerts an essential role in tumorigenesis, proliferation, migration, invasion, and inhibition of apoptosis, which means that *DUXAP8* acts as an oncogene in the occurrence and development of various malignant tumors (Jiang et al., 2019; Hu et al., 2020; Wang et al., 2020; Wei et al., 2020; Zhang et al., 2020; Guan et al., 2021). In addition, high-quality meta-analysis has been increasingly considered one of the keys and significant tools for achieving evidence (Yao et al., 2016; Tian et al., 2017; Li et al., 2018; Yang, 2018; Yang et al., 2018; Yan et al., 2019).

Thus, we performed this meta-analysis for the first time to explore the clinical prognostic role and functions of *DUXAP8* in human cancers. In addition, we employed data mining to investigate the prognostic value of *DUXAP8* in a range of tumor types to further validate our results. This study included an in-depth analysis of *DUXAP8* expression levels, as well as the relationship with tumor mutational burden (TMB), microsatellite instability (MSI), DNA methyltransferases (DNMTs), and mismatch repair (MMR).

## Materials and Methods

### Literature Search and Selection

We conducted a systematic search to identify relevant literature from its inception to 25 October 2021, including PubMed (Medline), Embase, and Cochrane Library. The retrieval words include: (“LINC *DUXAP8*” OR “LincRNA *DUXAP8*” OR “long non-coding RNA *DUXAP8*” OR “long noncoding RNA *DUXAP8*” OR “*DUXAP8* lncRNA”) and (“cancer” OR “carcinoma” OR “tumor” OR “tumor” OR “neoplasm” OR “adenoma” OR “sarcoma” OR “melanoma”). Additionally, we searched the reference lists of the primary literature and reviews to find pertinent supplementary literature.

### Inclusion and Exclusion Criteria

The inclusion criteria were: 1) articles to study the clinical functions of *DUXAP8* in different cancer tissues; 2) clinical trials in which patients were separated into two groups based on their *DUXAP8* expression levels; 3) studies that provided OS; 4) studies with sufficient data to generate HR and 95% confidence intervals (CI) or Kaplan-Meier curves; 5) case-control studies. The eliminated criteria included the following content: 1) studies on *DUXAP8*’s structure and functions; 2) nonhuman studies, reviews, editorials, specialist opinions, letters along with case reports; 3) studies having insufficient original data for survival analysis.

### Data Extraction and Quality Assessment

Two researchers independently assessed and obtained all the necessary data from the selected literature. The data extracted from each selected study are shown in Supplementary Table S1. If the relevant data were not directly accessible and only the Kaplan–Meier curves had been provided, we extracted the survival rates from the survival plot graphs and computed the HR, and the 95% CI indirectly (Parmar et al., 1998a; Parmar et al., 1998b). The Newcastle Ottawa Score (NOS) was used to evaluate the quality of the included studies (Stang, 2010). A NOS score of ≥6 indicates a high-quality study.

### Analysis of *DUXAP8* Expression in Cancer

UCSC Xena, derived from the TCGA database (https://xena.ucsc.edu/), provided us with data on 33 tumors, including RNA sequences, somatic mutations, clinicopathological characteristics, and survival rates. The cell line expression matrix was obtained from the CCLE dataset (https://portals.broadinstitute.org/ccle/about). We use the “Wilcox. test” to determine the difference in *DUXAP8* expression levels between tumor and normal tissues in various cancer types. Adrenocortical Carcinoma (ACC), Bladder Urothelial Carcinoma (BLCA), Breast invasive carcinoma (BRCA), Cervical squamous cell carcinoma and endocervical adenocarcinoma (CESC), Cholangiocarcinoma (CHOL), Colon adenocarcinoma (COAD), Lymphoid Neoplasm Diffuse Large B-cell Lymphoma (DLBC), Esophageal carcinoma (ESCA), Glioblastoma multiforme (GBM), Head and Neck squamous cell carcinoma (HNSC), Kidney Chromophobe (KICH), Kidney renal clear cell carcinoma (KIRC), Kidney renal papillary cell carcinoma (KIRP), Acute Myeloid Leukemia (LAML), Brain Lower Grade Glioma (LGG), Liver hepatocellular carcinoma (LIHC), Lung adenocarcinoma (LUAD), Lung squamous cell carcinoma (LUSC), Mesothelioma (MESO), Ovarian serous cystadenocarcinoma (OV), Pancreatic adenocarcinoma (PAAD), Pheochromocytoma and Paraganglioma (PCPG), Prostate adenocarcinoma (PRAD), Rectum adenocarcinoma (READ), Sarcoma (SARC), Skin Cutaneous Melanoma (SKCM), Stomach adenocarcinoma (STAD), Testicular Germ Cell Tumors (TGCT), Thyroid carcinoma (THCA), Thymoma (THYM), Uterine Corpus Endometrial Carcinoma (UCEC), Uterine Carcinosarcoma (UCS), Uveal Melanoma (UVM).

### Survival Analysis

We analyzed the relationships between *DUXAP8* expression and OS, disease-free interval (DFI), disease-specific survival (DSS), progression-free interval (PFI), age, and clinical stage. For survival analysis, the Kaplan-Meier method and COX analysis were used.

### Correlation of *DUXAP8* Expression With Tumor Mutational Burden, Microsatellite Instability, DNA Methyltransferases, and Mismatch Repair

TMB is defined as the total number of mutations per megabase of DNA. MSI is the spontaneous loss or gain of nucleotides from short tandem repeat DNA tracts. We utilized Spearman’s correlation analysis to explore the correlation between *DUXAP8* and TMB, MSI, the related genes of MMR, DNMTs, and immune checkpoint biomarkers. The resulting heatmap was implemented by using the R-packages “reshape2” and “RColorBrewer”.

### Pathway Analysis of *DUXAP8*

Downloaded gene sets from the Gene Set Enrichment Analysis (GSEA) website (https://www.gsea-msigdb.org/gsea/downloads.jsp) were used in the study. R-package “limma,” “org.Hs.eg.db,” “clusterProfiler,” and “enrichplot” were used to perform both Gene ontology (GO) and Kyoto Encyclopedia of Genes and Genomes (KEGG) of *DUXAP8* respectively.

### Data Synthesis and Statistical Analysis

The survival result was produced by employing information from the HR and the standard error (SE). During this meta-analysis, HRs were pooled by employing *I* ^2^ statistics to examine the heterogeneity of the applicable studies. The random-effects model was then used only if there was significant statistical heterogeneity between the studies (chi-squared test, *p* < 0.1, *I* ^2^ > 50%). We utilized the fixed-effects model (chi-squared test, *p* > 0.1, *I* ^2^ < 50%) where it was not applicable (Zhai et al., 2021). To show the meta-analysis outcomes, we employed forest plots. We used Begg’s test to see if there was any publication bias, and sensitivity analysis to see if the results were consistent. We applied STATA12.0, the R software to integrate and analyze the data. *p* < 0.05 was considered statistically significant.

## Results

### Studies Characteristics

Supplementary Figure S1 demonstrates the details concerning the screening process. A systematic search of the databases identified 114 studies published up to 25 October 2021. We excluded duplicate studies, studies irrelevant to the research subject, and studies that did not provide sufficient data. Therefore, there are a total of 25 studies that meet the final analysis conditions (Ma et al., 2017b; Xu et al., 2017; Lian et al., 2018; Lin et al., 2018; Du et al., 2019; Jiang et al., 2019; Chen et al., 2020a; Chen et al., 2020b; He et al., 2020; Hu et al., 2020; Nie et al., 2020; Wang et al., 2020; Wei et al., 2020; Yin et al., 2020; Zhang et al., 2020; Zhao et al., 2020; Chen et al., 2021a; Li et al., 2021a; Arabpour et al., 2021; Yang et al., 2021a; Chen et al., 2021b; Guan et al., 2021; Pang and Yang, 2021; Xing et al., 2021; Zhai et al., 2021). Moreover, the main features of the included studies have been summarized in Supplementary Table S1. The sample size of the 25 studies ranged between 31 and 522, with an average of 198.

All the included studies from 2016 to 2021 have been implemented and published in China except one study carried out in America. In total, 17 cancer types were included in our study: gastric cancer (GC) (Ma et al., 2017b), non-small cell lung cancer (NSCLC) (Yin et al., 2020; Chen et al., 2021a), cervical cancer (CC) (Chen et al., 2020b), oral cancer (OC) (Chen et al., 2020a), colorectal cancer (CRC) (Du et al., 2019; He et al., 2020), papillary thyroid carcinoma (PTC) (Pang and Yang, 2021), LGG (Zhao et al., 2020), PAAD (Lian et al., 2018), hepatocellular carcinoma (HCC) (Jiang et al., 2019; Hu et al., 2020; Wang et al., 2020; Wei et al., 2020; Zhang et al., 2020; Guan et al., 2021), acute myeloid Leukemia (AML) (Zhai et al., 2021), BLCA (Lin et al., 2018), KIRC (Xing et al., 2021), neuroblastoma (Nie et al., 2020), ovarian cancer (Li et al., 2021a), osteosarcoma (Yang et al., 2021a), renal cell carcinoma (RCC) (Xu et al., 2017), melanoma (Chen et al., 2021b), breast cancer (BC) (Arabpour et al., 2021).

### Correlation of the *DUXAP8* Expression Level With the Overall Survival

**FIGURE 1:**
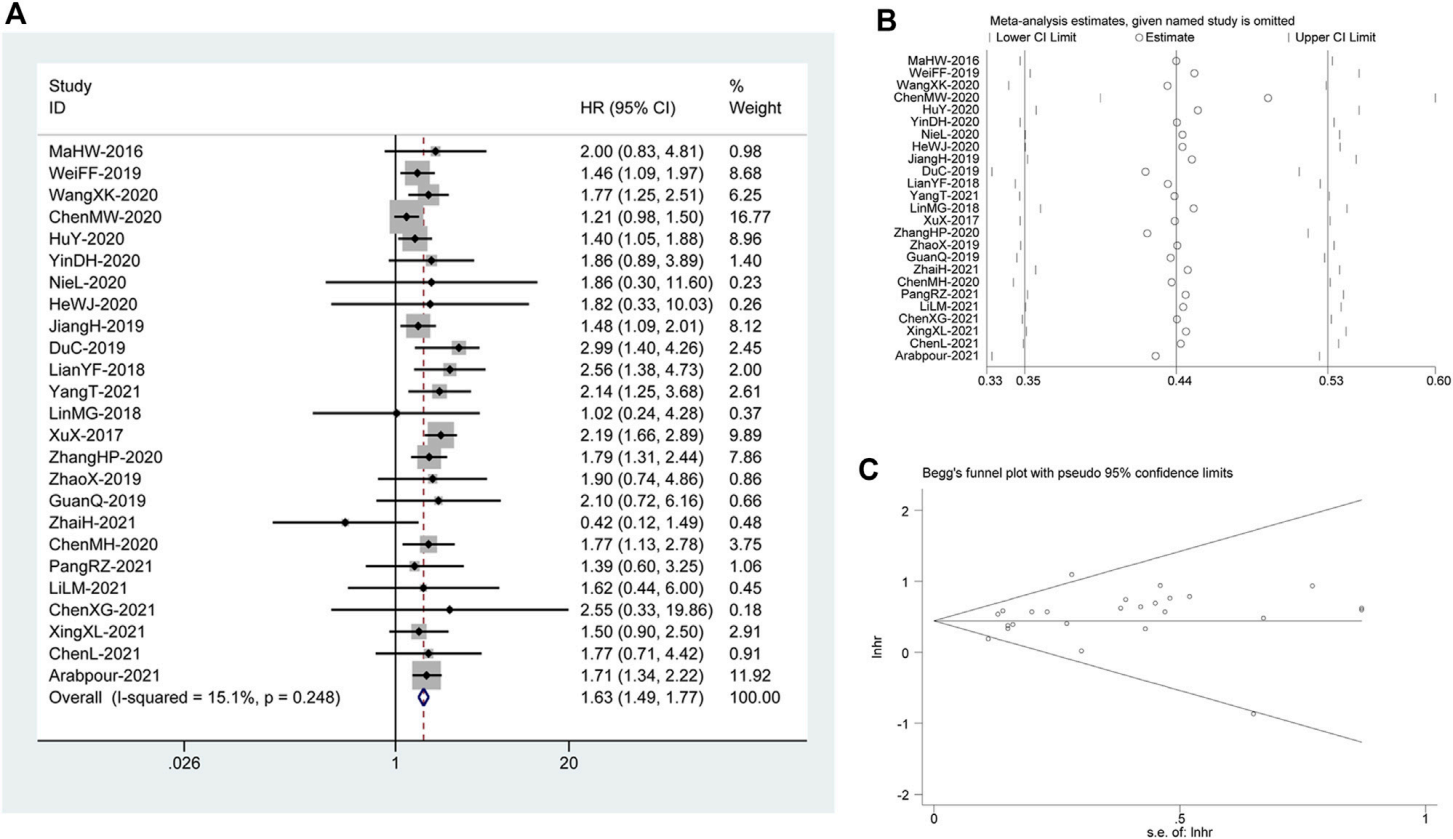
**(A)** Forest plot reflecting the association between OS and *DUXAP8* expression level in cancer. **(B)** Sensitivity analysis for studies about OS. **(C)**: Begg’s funnel plot of *DUXAP8* for overall survival.

There were 25 studies (Ma et al., 2017b; Xu et al., 2017; Lian et al., 2018; Lin et al., 2018; Du et al., 2019; Jiang et al., 2019; Chen et al., 2020a; Chen et al., 2020b; He et al., 2020; Hu et al., 2020; Nie et al., 2020; Wang et al., 2020; Wei et al., 2020; Yin et al., 2020; Zhang et al., 2020; Zhao et al., 2020; Chen et al., 2021a; Li et al., 2021a; Arabpour et al., 2021; Yang et al., 2021a; Chen et al., 2021b; Guan et al., 2021; Pang and Yang, 2021; Xing et al., 2021; Zhai et al., 2021), consisting of 4,757 patients, included for OS analysis. A correlation analysis has been performed to explore between *DUXAP8* and the poor OS in patients diagnosed with cancer. It applied the fixed effect model to the studies (*I* ^2^ = 15.1%, *P*_Q=_ 0.248). As illustrated in Figure 1A, there was a pooled HR = 1.63 between *DUXAP8* and the OS (95% CI, 1.49–1.77, *p* < 0.001), revealing significantly worse OS in the cancer patients with high expression of *DUXAP8*.

### Publication Bias and Sensitivity Analysis

We constructed Begg’s funnel plot to assess publication bias among the reviews. There was no indication of noticeable OS disparity (*p*>|t| = 0.164; Figure 1C). In addition, we ran a sensitivity analysis after discarding each paper to confirm the validity of the relationship between *DUXAP8* expression and OS. This analysis showed no significant change in the results. Therefore, the meta-analysis results were trustworthy. (Figure 1B).

### Subgroup Analysis of the Relationship Between *DUXAP8* Expression Level and Overall Survival

**FIGURE 2:**
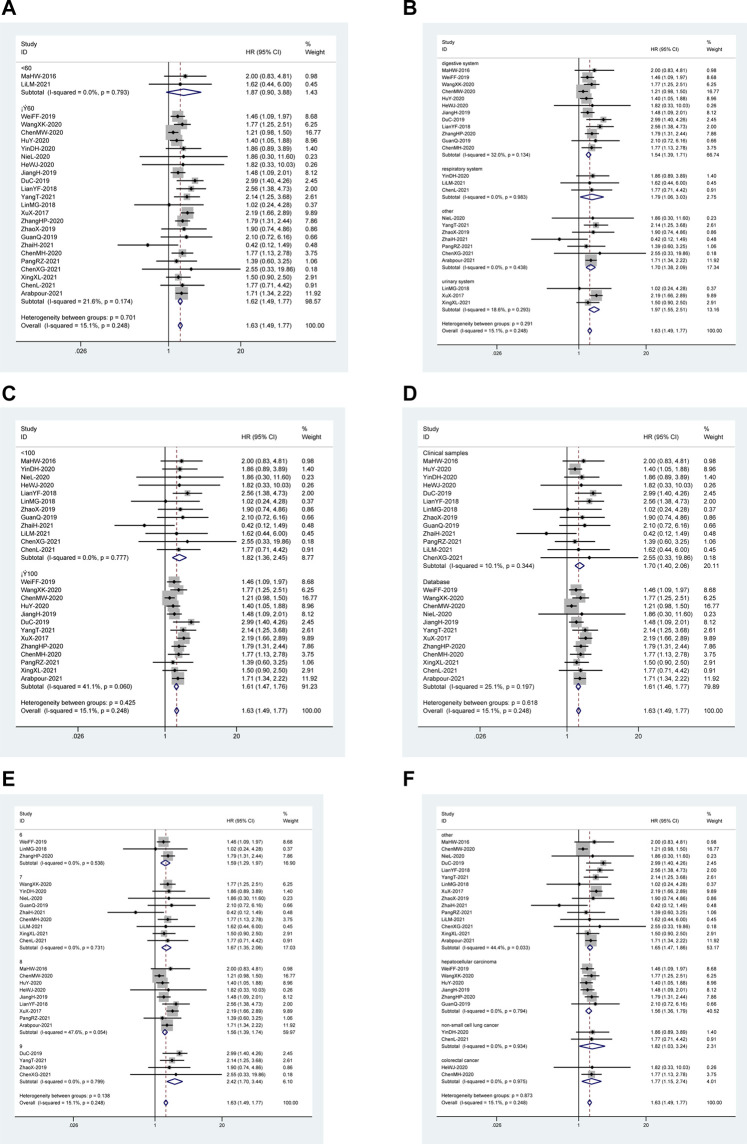
Forest plot reflecting the association between OS and lncRNA *DUXAP8* expression level in cancer. **(A)**: Subgroup analysis stratified by follow-up time. **(B)**: Subgroup analysis stratified by the system of cancer. **(C)**: Subgroup analysis stratified by sample size. **(D)**: Subgroup analysis stratified by sample source. **(E)**: Subgroup analysis stratified by NOS score. **(F)**: Subgroup analysis stratified by type of cancer.

Based on the following factors, subgroup analysis was done to evaluate the relationship between *DUXAP8* expression levels and OS: follow-up time (<60 or ≥60 months) (Figure 2A), the system of cancer (digestive system, urogenital system, respiratory system, hematologic system or other) (Figure 2B), sample size (<100 or ≥100 tissues) (Figure 2C), sample source (clinical samples or database) (Figure 2D), the quality of included literature (NOS scores) (Figure 2E), and type of cancer (Figure 2F). In these malignancies, the outcomes of the subgroup analysis didn’t change the predictive value of *DUXAP8* for OS.

### Multifaceted Prognostic Value of *DUXAP8* in Pan-Cancers

**FIGURE 3:**
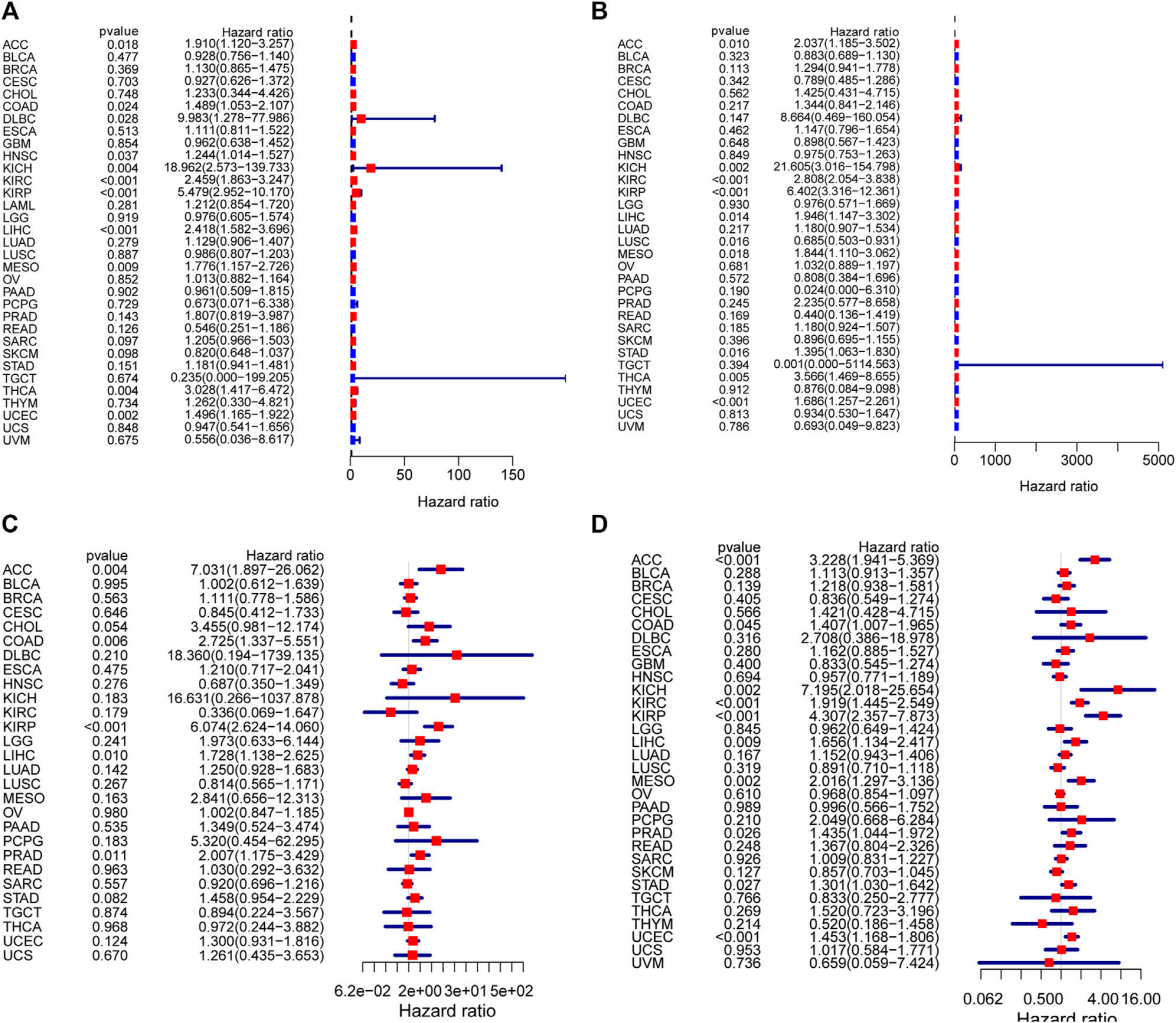
Correlation analysis of *DUXAP8* expression with survival using the COX method for different types of cancers in TCGA. **(A)**: OS. **(B)**: DSS. **(C)**: DFI. **(D)**: PFI.

To assess *DUXAP8*’s ability to predict pan-cancer, we evaluated multiple datasets. We used COX analysis to evaluated the *DUXAP8*-related survival (OS, DSS, DFI, and PFI) (Figure 3). Thus, we discovered that *DUXAP8* was a detrimental factor in ACC (OS: HR = 1.910; DSS: HR = 2.037; DFI: HR = 7.031; PFI: HR = 3.228), LIHC (OS: HR = 2.418; DSS: HR = 1.946; DFI: HR = 1.728; PFI:HR = 1.656), KIRP (OS: HR = 5.479; DSS: HR = 6.402; DFI: HR = 6.074; PFI:HR = 4.307), KIRC (OS: HR = 2.459; DSS: HR = 2.808; PFI:HR = 1.919), UCEC (OS: HR = 1.496; DFI: HR = 1.686, *p* < 0.001; PFI:HR = 1.453), KICH (OS: HR = 18.962; DSS: HR = 21.605; PFI:HR = 7.195), MESO (OS: HR = 1.776; DSS: HR = 1.844; PFI:HR = 2.016), COAD (OS: HR = 1.489; DFI: HR = 2.725; PFI:HR = 1.407), THCA (OS: HR = 3.028; DSS: HR = 3.566), STAD (DSS: HR = 1.395; PFI: HR = 1.301), PRAD (DFI: HR = 2.007; PFI: HR = 1.435), DLBC (OS: HR = 9.983), and HNSC (OS: HR = 1.244).

We next used Kaplan-Meier method to investigate the *DUXAP8*-related survival in the TCGA (Supplementary Figure S2). We discovered that low levels of *DUXAP8* expression were associated with a poor prognosis, which included SKCM (OS: *p* = 0.003; DSS: *p* = 0.033; PFI: *p* = 0.002), READ (DSS: *p* = 0.041), LGG (PFI: *p* = 0.036). Conversely, high levels of *DUXAP8* expression were associated with a poor prognosis in KIRC (OS: *p* < 0.001; DSS: *p* < 0.001; PFI: *p =* 0.001), KIRP (OS: *p <* 0.001; DSS: *p* = 0.001; PFI: *p =* 0.015), LIHC (OS: *p =* 0.001; DFI: *p =* 0.041; PFI: *p =* 0.020), UCEC (OS: *p =* 0.007; DSS: *p =* 0.022; PFI: *p =* 0.034), ACC (DSS: *p =* 0.028; PFI: *p <* 0.001), BRCA (DSS: *p* = 0.048), STAD (DSS: *p =* 0.033, COAD (DFI: *p =* 0.019), PRAD (DFI: *p =* 0.019), and MESO (PFI: *p =* 0.034).

**FIGURE 4:**
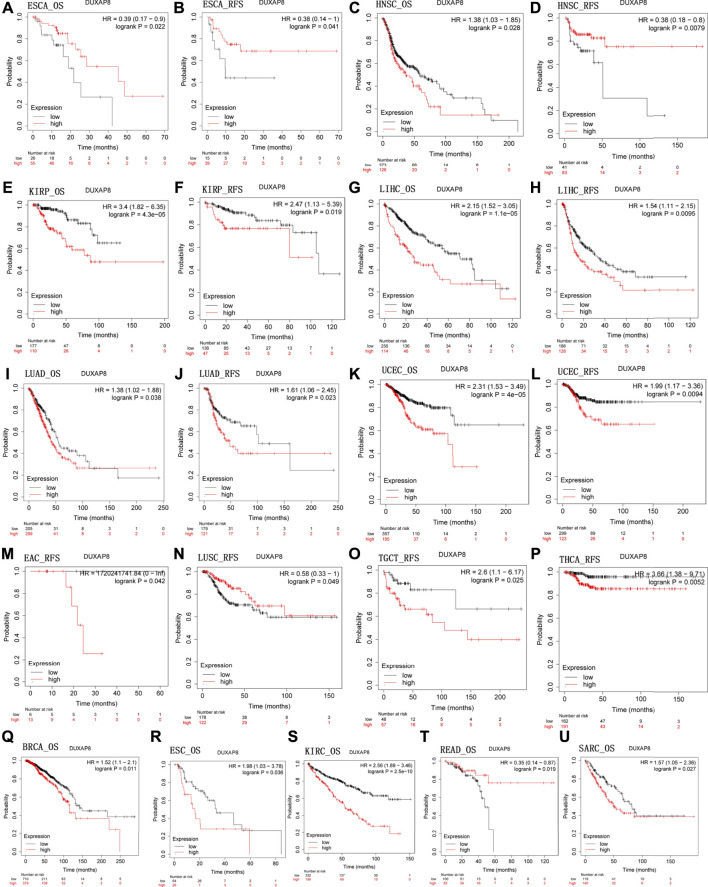
Kaplan-Meier survival curves comparing the high and low expression of *DUXAP8* gene in various cancer types in Kaplan-Meier Plotter. OS and RFS of **(A,B)** ESCA, **(C,D)** HNSC, **(E,F)** KIRP, **(G,H)** LIHC, **(I,J)** LUAD, **(K,L)** UCEC. RFS of **(M)** EAC, **(N)** LUSC, **(O)** TGCT, and **(P)** THCA. OS of **(Q)** BRCA, **(R)** ESC, **(S)** KIRC, **(T)** READ, and **(U)** SARC. OS, overall survival; RFS, relapse-free survival.

We next used Kaplan-Meier plotter (https://kmplot.com/analysis) to evaluate *DUXAP8*-related survival (OS and RFS). Interestingly, we were able to verify that *DUXAP8* had a protective prognostic role in ESCA (OS: HR = 0.39; RFS, HR = 0.38) (Figures 4A,B), and READ (OS: HR = 0.35) (Figure 4T). In contrast, *DUXAP8* expression had a detrimental effect in HNSC (OS: HR = 1.38) (Figure 4C), KIRP (OS: HR = 3.4; RFS, HR = 2.47) (Figures 4E,F), LIHC (OS: HR = 2.15; RFS, HR = 1.54) (Figures 4G,H), LUAD (OS: HR = 1.38; RFS, HR = 1.61) (Figures 4I,J), UCEC (OS: HR = 2.31; RFS, HR = 199) (Figures 4K–L), BRCA (OS: HR = 1.52) (Figure 4Q), ESC (OS: HR = 1.98) (Figure 4R), KIRC (OS: HR = 2.56) (Figure 4S), SARC (OS: HR = 1.57) (Figure 4U). *DUXAP8* expression was significantly correlated with patients’ RFS in LUSC, EAC, TGCT, HNSC, and THCA.

### Correlation Analysis of *DUXAP8* Expression and Clinicopathology

**FIGURE 5:**
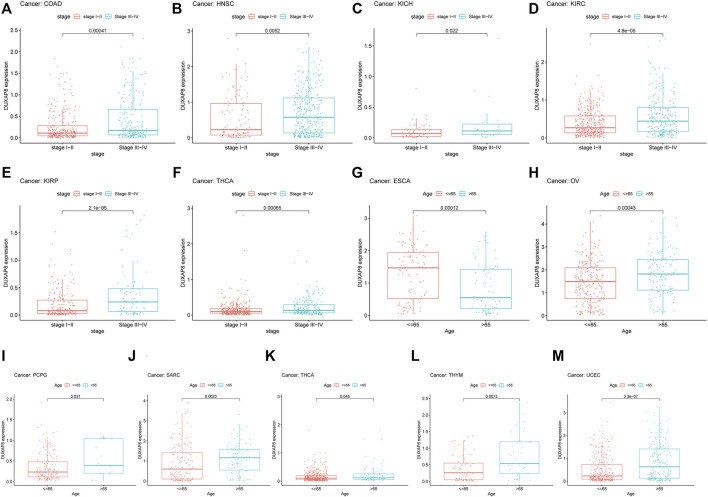
Relationship between the *DUXAP8* gene expression and clinicopathological features of Pan-cancer. *DUXAP8* gene expression is related to the stage in COAD **(A)**, HNSC **(B)**, KICH **(C)**, KIRC **(D)**, KIRP **(E)**, and YHCA **(F)**. *DUXAP8* gene expression is associated with age in ESCA **(G)**, OV **(H)**, PCPG **(I)**, SARC **(J)**, THCA **(K)**, THYM **(L)**, and UCEC **(M)**.

*DUXAP8* expression has been linked to numerous malignancies’ clinicopathological characteristics (Figure 5). Concerning COAD, HNSC, KICH, KIRC, KIRP, and THCA (Figures 5A–F), *DUXAP8* was highly expressed in stages III-IV. In particular, patients over the age of 65 had greater *DUXAP8* expression in OV, PCPG, SARC, THCA, THYM, and UCEC (Figures 5H–M). *DUXAP8* was, on the other hand, highly expressed in individuals under the age of 65, notably in ESCA patients (Figure 5G).

### Expression of *DUXAP8* in Pan-Cancers

**FIGURE 6:**
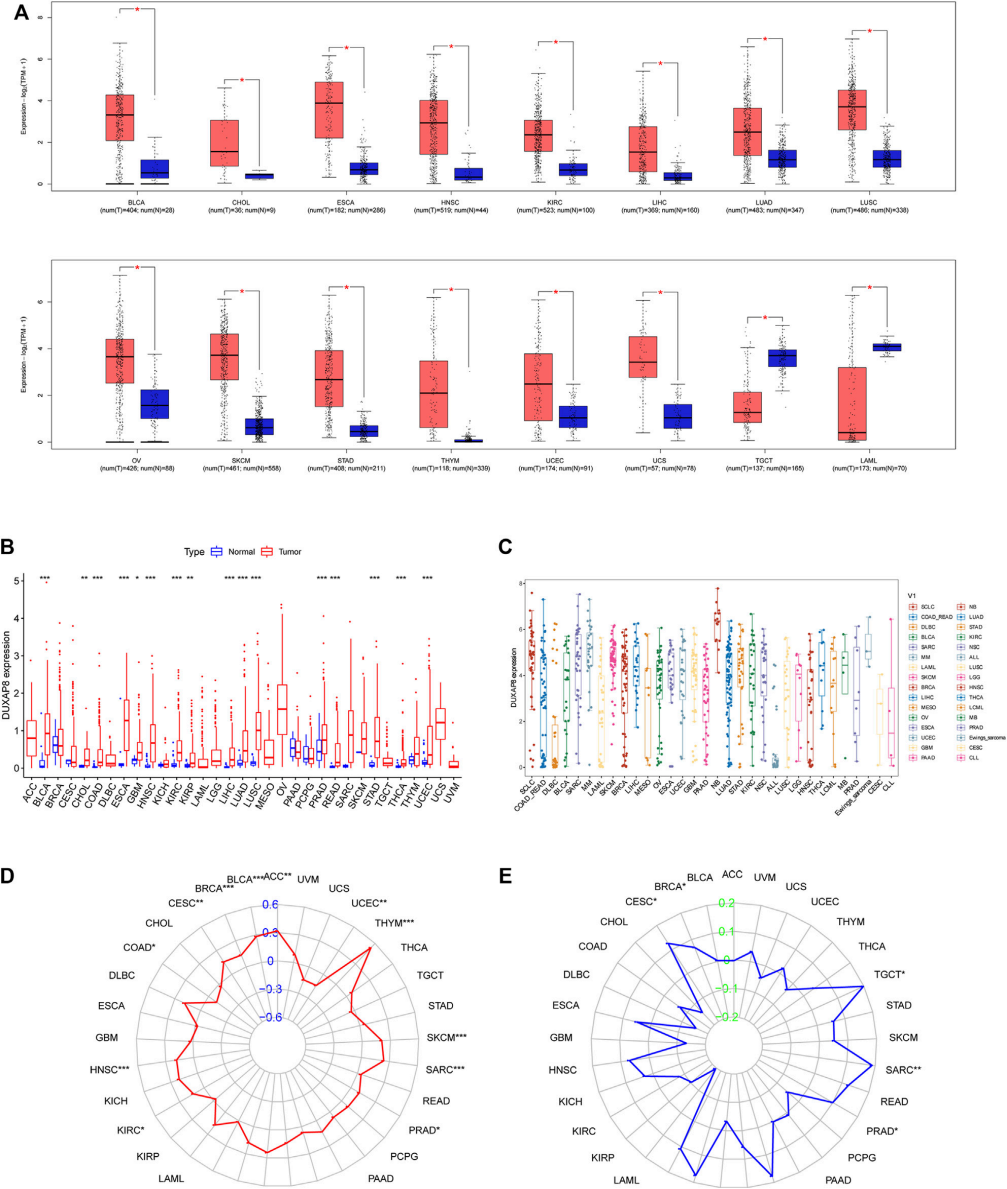
**(A)** *DUXAP8* expression levels in different cancer types from TCGA data by Gene Expression Profiling Interactive Analysis (GEPIA). **(B)** *DUXAP8* expression levels in different cancer types from TCGA data. The red fusiformis represents tumor tissue and the blue fusiformis represents normal tissue. *p* < 0.05, <0.01, <0.001 are represented by “*”, “**”, “***” respectively. **(C)** The expression distribution of *DUXAP8* in different tumor tissues. **(D)** The radar chart illustrated the association between TMB and *DUXAP8* expression in different cancers. **(E)** The radar chart illustrated the relationship between MSI and *DUXAP8* expression in different cancers. The blue curve represents the correlation coefficient, and the green value represents the range.

We first used GEPIA to investigate *DUXAP8* expression in pan-cancer from the TCGA and GTEx databases. *DUXAP8* was shown to be highly expressed in BLCA, CHOL, ESCA, HNSC, KIRC, LIHC, UAD, LUSC, OV, SKCM, STAD, THYM, UCEC, and UCS, except for TGCT and LAML, where it was found to be weakly expressed (Figure 6A). Data from the TCGA showed that *DUXAP8* was significantly higher in BLCA, CHOL, COAD, ESCA, GBM, HNSC, KIRC, KIRP, LIHC, LUAD, LUSC, PRAD, READ, STAD, THCA, and UCEC (Figure 6B). Figure 6C represents the relative amounts of *DUXAP8* expression in several cell lines based on CCLE data.

### Correlation of *DUXAP8* Expression With Tumor Mutational Burden, Microsatellite Instability, DNA Methyltransferases, and Mismatch Repair

**FIGURE 7:**
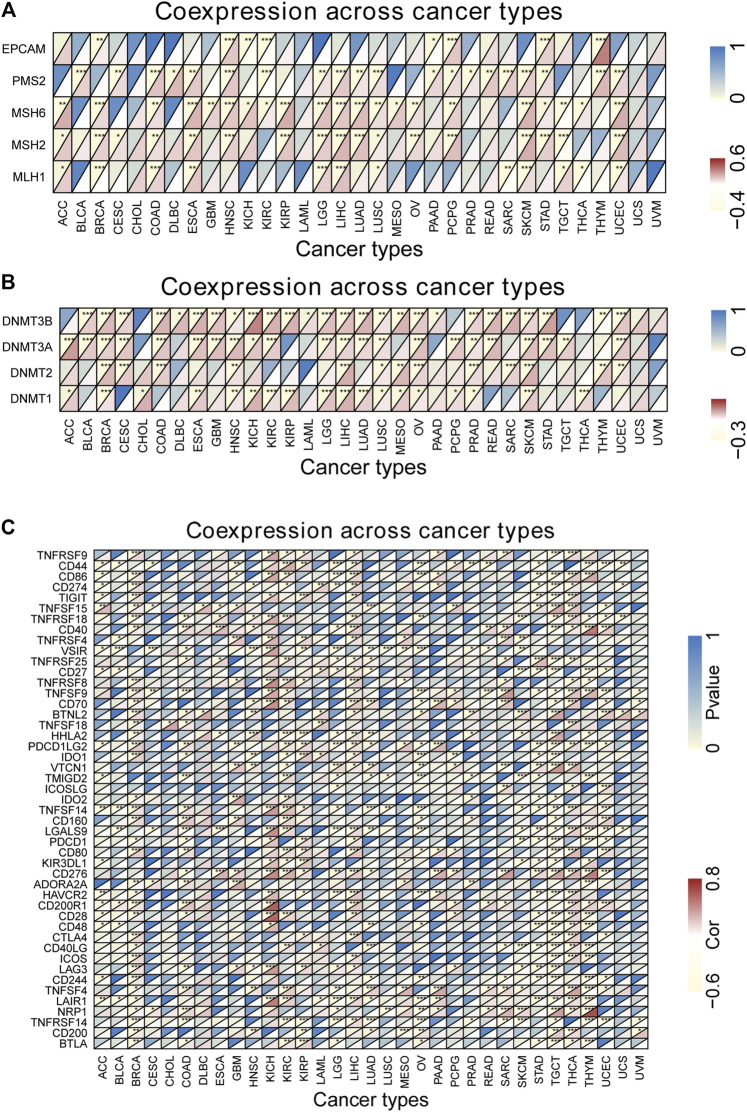
Co-expression analysis between DUXAP8 expression and five mismatch repair genes **(A)**, DNA methyltransferase **(B)** and immune genes **(C)** in cancers. *p < 0.05; **p < 0.01; ***p < 0.001. Cor, correlation coefficient. The horizontal axis represents cancer types, the vertical axis represents immune genes, and each small rectangular module represents the co-expression of the gene and DUXAP8 in cancer, during them, the upper left corner asterisk and color represent the P-value, and the lower right corner color represents the Cor. *p < 0.05; **p < 0.01; ***p < 0.001. Cor, correlation coefficient.

We found that *DUXAP8* expression was positively correlated with the TMB in THYM, BLCA, LUAD, SKCM, BRCA, HNSC, SARC, LIHC, LUSC, ACC, CESC, KIRC, PRAD, OV, while negatively correlated with the TMB in UCEC, COAD (Figure 6D). Moreover, *DUXAP8* expression was found to be positively correlated to the MSI in LIHC, SARC, TGCT, LGG, BRCA, PRAD, and CESC (Figure 6E). In 29 of the 33 cancer types, TIGIT was correlated with the expression of at least one MMR-related gene (Figure 7A). *DUXAP8* expression was positively correlated with DNMTs expression level in most tumors (Figure 7B). *DUXAP8* expression was correlated with immune checkpoint biomarkers in most tumors, especially in BRCA, COAD, KRCH, KIRC, KIRP, LIHC, STAD, TGCT, THCA, and THYM (Figure 7C).

### Pathway Analysis in Pan-Cancers

**FIGURE 8:**
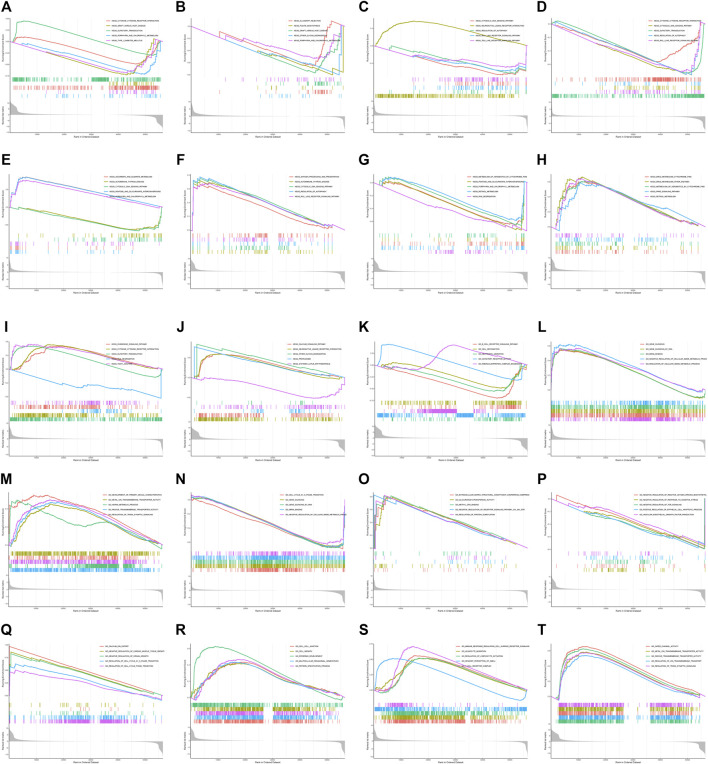
**(A–J)** GO functional annotation of *DUXAP8* gene in ACC, BLCA, HNSC, LUSC, OV, PAAD, PCPG, TGCT, THCA, and UVM. **(K–T)** KEGG pathway analysis of *DUXAP8* gene ACC, BLCA, HNSC, LUSC, OV, PAAD, PCPG, TGCT, THCA, and UVM. Different color curves indicate that *the DUXAP8* gene regulated different functions or pathways of different cancers, with peaks of curves upward indicating positive regulation and peaks of curves downward representing negative regulation.

To investigate the biological function and KEGG pathway of *DUXAP8* expression in pan-cancers, we conducted GESA (Figure 8). The results of GO indicated that *DUXAP8* was able to regulate the cell cycle, cell junction, cell recognition, cell growth, negative regulation of cellular amide metabolic process, gene silencing, and mRNA binding. The results demonstrate that *DUXAP8* expression is associated with several pathways: pentose and glucuronate interconversions, porphyrin and chlorophyll metabolism, retinol metabolism, cytokine receptor interaction, RNA degradation, and regulation of autophagy.

## Discussion

Great improvements have been achieved in cancer detection and treatment. However, the 5-year survival rate remains relatively low for most cancers. Human health is seriously threatened by cancer. Some lncRNAs have the potential to serve as biomarkers for diagnosing and monitoring tumors due to their specific expression during tumor occurrence and development (Qi and Du, 2013). *DUXAP8* is significantly overexpressed in cancer tissues compared to nearby non-tumor tissues, according to observations (Ma et al., 2017b; Du et al., 2019; Chen et al., 2020a; Chen et al., 2020b; He et al., 2020; Yin et al., 2020; Chen et al., 2021a). Thus, we first conducted this meta-analysis to examine if there was a correlation between lncRNA *DUXAP8* expression and overall survival in order to better evaluate its predictive potential. Our study revealed a significantly worse OS in cancer patients with high expression of *DUXAP8*. For this, we concluded that high levels of *DUXAP8* expression are associated with a poor prognosis for cancer patients and that *DUXAP8* may be a predictor of poor prognosis in cancer patients.

In addition, we employed data mining to investigate the prognostic value of *DUXAP8* in a range of tumor types to further validate our results. To assess *DUXAP8*’s ability to predict pan-cancer, we evaluated multiple datasets. Cox regression model discovered that *DUXAP8* was a detrimental factor in ACC, COAD, DLBC, HNSC, KICH, KIRC, KIRP, LIHC, MESO, THCA, and UCEC in the TCGA. Even more, we discovered that high levels of *DUXAP8* expression were associated with a poor prognosis in ESCA, HNSC, KIRP, LIHC, LUAD, UCEC, BRCA, ESC, KIRC, READ, and SARC, but with a good prognosis in ESCA, SKCM and READ by Kaplan-Meier method.

We found that *DUXAP8* expression was positively correlated with the TMB in THYM, BLCA, LUAD, SKCM, BRCA, HNSC, SARC, LIHC, LUSC, ACC, CESC, KIRC, PRAD, OV, while negatively correlated with the TMB in UCEC, COAD (Figure 6D). Moreover, *DUXAP8* expression was found to be positively correlated to the MSI in LIHC, SARC, TGCT, LGG, BRCA, PRAD, and CESC (Figure 6E). *DUXAP8* expression was correlated with immune checkpoint biomarkers in most tumors, especially in BRCA, COAD, KRCH, KIRC, KIRP, LIHC, STAD, TGCT, THCA, and THYM. *DUXAP8* expression was positively correlated with MMR-related genes level in most tumors.

This study included an in-depth analysis of *DUXAP8* expression levels, as well as the relationship with TMB, MSI, MMR, DNMTs, and immune checkpoint biomarkers in 33 cancer types. This study found that the expression of *DUXAP8* is significantly correlated with TMB in seven cancer types and MSI in seven cancer types. *DUXAP8* expression was positively correlated with MMR-related genes level in most tumors. The research suggested that *DUXAP8* expression may have an effect on cancer patients’ response to immune checkpoint therapy, which will benefit the further understanding of immunotherapy’s molecular mechanism in cancer treatment.

*DUXAP8* was significantly higher in BLCA, CHOL, ESCA, HNSC, KIRC, LIHC, UAD, LUSC, OV, SKCM, STAD, THYM, UCEC, and UCS, except for TGCT and LAML, where it was found to be weakly expressed. *DUXAP8* was significantly higher in BLCA, CHOL, COAD, ESCA, GBM, HNSC, KIRC, KIRP, LIHC, LUAD, LUSC, PRAD, READ, STAD, THCA, and UCEC in the TCGA and GTEx databases. There is accumulating evidence to reveal that *DUXAP8* is aberrantly expressed in several malignancies and appears to contribute to the development and progression of multiple cancers, including GC (Ma et al., 2017b), NSCLC (Yang et al., 2019; Ji et al., 2020; Yin et al., 2020; Chen et al., 2021a; Liu et al., 2021a), CC (Chen et al., 2020b), OC (Chen et al., 2020a), CRC (Du et al., 2019; Gong et al., 2019; He et al., 2020; Liang et al., 2021), PTC (Liu et al., 2021b; Pang and Yang, 2021), LGG (Zhao et al., 2020), PC (Lian et al., 2018; Li et al., 2021b), HCC (Jiang et al., 2019; Hu et al., 2020; Wang et al., 2020; Wei et al., 2020; Zhang et al., 2020; Guan et al., 2021), AML (Zhai et al., 2021), BLCA (Jiang et al., 2018; Lin et al., 2018), NB (Nie et al., 2020), ovarian cancer (Meng et al., 2020; Li et al., 2021a), osteosarcoma (Yang et al., 2021a), RCC (Xu et al., 2017; Huang et al., 2018; Xing et al., 2021), melanoma (Chen et al., 2021b), BC (Arabpour et al., 2021; Yang et al., 2021b), esophageal carcinoma (Liu et al., 2018). This is basically consistent with this study.

Although many studies found that lncRNA *DUXAP8* serves as an important prognostic factor for patients with a variety of tumors, the underlying systems of how the lncRNA *DUXAP8* impacts cancer are still unknown. *DUXAP8*-related molecular targets, proteins, pathways, and noncoding RNA (microRNAs and circRNAs) were methodically described in this meta-analysis to provide a reference for mechanistic exploration into the carcinogenesis function of *DUXAP8* in various cancers (Supplementary Table S2). *DUXAP8* induced an EMT phenotype transition and epigenetic alteration *via* various signaling pathways covering pathways of *Wnt/β-catenin* (Zhai et al., 2021)in the AML, *miR-126-5p/PTEN/PI3K/AKT* (Jiang et al., 2018; Lin et al., 2018) in the BLCA, *miR-130a-3p* (Arabpour et al., 2021; Yang et al., 2021b) in the BC, EZH2 (Ma et al., 2017b; Lian et al., 2018; Du et al., 2019; Gong et al., 2019; Chen et al., 2020a; He et al., 2020) in the CRC, OC and GC, miR-590-5p (Jiang et al., 2019; Hu et al., 2020; Meng et al., 2020; Wang et al., 2020; Wei et al., 2020; Zhang et al., 2020; Li et al., 2021a; Guan et al., 2021) in the ovarian cancer and HCC, *miR-126* (Xu et al., 2017; Huang et al., 2018; Xing et al., 2021) in the RCC, *miR-3182/NUPR1* (Chen et al., 2021b) in the melanoma, *miR-409-3p/HK2/LDHA* (Yang et al., 2019; Ji et al., 2020; Yin et al., 2020; Chen et al., 2021a; Liu et al., 2021a) in the NSCLC, *miR-448/WTAP/Fak* (Lian et al., 2018; Li et al., 2021b) in the PC, *miR-223-3p* (Liu et al., 2021b; Pang and Yang, 2021) in the PTC, miR-29 (Nie et al., 2020) in the neuroblastoma, *miR-635/TOP2A* (Yang et al., 2021a) in the osteosarcoma.

Nonetheless, there were several limitations to this meta-analysis. First, only 25 studies with several types of tumors were included in the meta-analysis, so the results need to be further confirmed in a large cohort in the future. Second, it might not be precise enough to calculate HRs and corresponding 95% CIs through survival curves in the place of precisely obtaining them from the primary publications. Third, all included studies divided the cut-off values for high and low lncRNA *DUXAP8* expression by inconsistent methods, which made the data less accurate. Fourth, patients from China made up the majority of the eligible trials, which means that they may not accurately represent all cancer patients worldwide. For future clinical trials, it is imperative that high-quality, multi-center studies with a larger sample size be done to confirm and reinforce our preliminary findings.

## Conclusion

This study revealed that *DUXAP8* might serve as a prognostic biomarker and potential therapeutic target for cancer. It can be used to improve cancer diagnosis, discover potential treatment targets, and improve prognosis. Therefore, combining regular clinical examinations with an evaluation of *DUXAP8* expression provides individuals with a targeted prognosis and more treatment options.

## Data Availability

The original contributions presented in the study are included in the article/Supplementary Material, further inquiries can be directed to the corresponding author.

## Supplementary Material

Supplementary Figure S1 Flowchart showing the steps of study selection in this meta-analysis.

Supplementary Figure S2 Kaplan-Meier survival curves comparing high and low expression of *DUXAP8* for different cancer types. **(A)**: OS of KIRC, KIRP, LIHC, SKCM, UCEC. **(B)** DSS of ACC, BRCA, KIRC, KIRP, READ, SKCM, STAD, and UCEC. **(C)**: DFI of COAD, LIHC, PRAD. **(D)**: PFI of ACC, KIRC, KIRP, LGG, LIHC, MESO, SKCM, and UCEC.
