# Supplementary material for: LncRNA DUXAP8 as a prognostic biomarker for various cancers: A meta-analysis and bioinformatics analysis
Source: Front Genet. 2022 Aug 15;13:907774. doi: 10.3389/fgene.2022.907774 (PMC9420988; doi:10.3389/fgene.2022.907774)
Supplement: Supplementary file 1 [file Table1.DOCX]

| **Study** | **Region** | **Tumor type** | **Study population (high/low)** | **Sample size** | **follow up months** | **Sample type** | **Cut off** | **RNA** | **Test method** | **Outecome measure** | **hazard ratios** | **NOS score** |
| --- | --- | --- | --- | --- | --- | --- | --- | --- | --- | --- | --- | --- |
| MaHW-2016 | China | GC | 36/36 | 72 | 40 | tissue | Median | DUXAP8 | qRT-PCR | OS | K-M | 8 |
| WeiFF-2019 | China | HCC | 182/182 | 364 | 120 | tissue | NA | DUXAP8 | qRT-PCR | OS | K-M | 6 |
| WangXK-2020 | China | HCC | 185/185 | 370 | 133 | tissue | NA | DUXAP8 | qRT-PCR | OS | K-M | 7 |
| ChenMW-2020 | China | OC | 259/259 | 518 | 200 | tissue | NA | DUXAP8 | qRT-PCR | OS | K-M | 8 |
| HuY-2020 | China | HCC | 182/182 | 364 | 120 | tissue | NA | DUXAP8 | qRT-PCR | OS | K-M | 8 |
| YinDH-2020 | China | NSCLC | 33/33 | 66 | 60 | tissue | NA | DUXAP8 | qRT-PCR | OS | K-M | 7 |
| NieL-2020 | China | NB | 23/22 | 45 | 60 | tissue | Median | DUXAP8 | qRT-PCR | OS | K-M | 7 |
| HeWJ-2020 | China | CRC | 15/15 | 30 | 300 | tissue | NA | DUXAP8 | qRT-PCR | OS | K-M | 8 |
| JiangH-2019 | China | HCC | 182/182 | 364 | 120 | tissue | NA | DUXAP8 | qRT-PCR | OS | K-M | 8 |
| DuC-2019 | China | CRC | 64/63 | 127 | 60 | tissue | NA | DUXAP8 | qRT-PCR | OS | K-M | 9 |
| LianYF-2018 | China | PDAC | 29/29 | 58 | 80 | tissue | Median | DUXAP8 | qRT-PCR | OS | K-M | 8 |
| YangT-2021 | China | OS | 66/66 | 132 | 200 | tissue | Mean | DUXAP8 | qRT-PCR | OS | K-M | 9 |
| LinMG-2018 | China | BLCA | 16/15 | 31 | 100 | tissue | NA | DUXAP8 | qRT-PCR | OS | K-M | 6 |
| XuX-2017 | China | RCC | 261/261 | 522 | 120 | tissue | NA | DUXAP8 | qRT-PCR | OS | K-M | 8 |
| ZhangHP-2020 | China | HCC | 136/234 | 370 | 120 | tissue | NA | DUXAP8 | qRT-PCR | OS | K-M | 6 |
| ZhaoX-2019 | China | LGG | 28/30 | 58 | 80 | tissue | Mean | DUXAP8 | qRT-PCR | OS | K-M | 9 |
| GuanQ-2019 | China | HCC | 19/19 | 38 | 60 | tissue | NA | DUXAP8 | qRT-PCR | OS | K-M | 7 |
| ZhaiH-2021 | China | AML | 20/20 | 40 | 84 | tissue | NA | DUXAP8 | qRT-PCR | OS | K-M | 7 |
| ChenMH-2020 | China | CC | 135/134 | 269 | 150 | tissue | log2-fold | DUXAP8 | qRT-PCR | OS | K-M | 7 |
| PangRZ-2021 | China | PTC | 255/252 | 507 | 150 | tissue | NA | DUXAP8 | qRT-PCR | OS | K-M | 8 |
| LiLM-2021 | China | OCa | NA | 33 | 40 | tissue | NA | DUXAP8 | qRT-PCR | OS | K-M | 7 |
| ChenXG-2021 | China | SKCM | 22/21 | 43 | 60 | tissue | Median | DUXAP8 | qRT-PCR | OS | K-M | 9 |
| XingXL-2021 | China | KIRC | 132/133 | 265 | 200 | tissue | NA | DUXAP8 | qRT-PCR | OS | K-M | 7 |
| ChenL-2021 | China | NSCLC | NA | 515 | 266 | tissue | Mean | DUXAP8 | qRT-PCR | OS | K-M | 7 |
| Arabpour-2021 | Iran | BC | 299/301 | 600 | 250 | tissue | Mean | DUXAP8 | qRT-PCR | OS | K-M | 7 |

Supplementary Table 1: Summary of included eligible studies for meta-analysis in the present study
