# Supplementary material for: LncRNA DUXAP8 as a prognostic biomarker for various cancers: A meta-analysis and bioinformatics analysis
Source: Front Genet. 2022 Aug 15;13:907774. doi: 10.3389/fgene.2022.907774 (PMC9420988; doi:10.3389/fgene.2022.907774)
Supplement: Supplementary file 2 [file Table2.DOCX]

| Acute Myeloid Leukemia | downregulate | Cell proliferation | Wnt/β-catenin | [23] |
| --- | --- | --- | --- | --- |
| Bladder Urothelial Carcinoma | upregulate | Cell proliferation | PTEN | [13, 33] |
| Breast cancer | upregulate | Cell proliferation | LINC00963，miR-130a-3p，SAPCD2 | [1, 36] |
| Colorectal Cancer | upregulate | Cell proliferation, invasion, migration, EMT | EZH2, miR-519b/ZNF277, miR-577 | [6, 8, 29, 30] |
| Cervical cancer | upregulate | Cell proliferation |  | [3] |
| Epithelial Ovarian Cancer | upregulate | Cell proliferation, migration | microRNA-29a-3p, miR-590-5p | [11, 34] |
| Esophageal carcinoma | upregulate | Cell proliferation, invasion |  | [37] |
| gastric carcinoma | upregulate | Cell proliferation | EZH2， SUZ12/ PLEKHO1 | [14] |
| hepatocellular carcinoma | upregulate | Cell proliferation, invasion, migration | miR-422a, miR-485-5p, KLF2, MiR-490-5p, miR-9-3p/IGF1R | [7, 9, 10, 17, 18, 24] |
| renal cell carcinoma | upregulate | Cell invasion | miR-126 | [19, 20, 35] |
| Brain Lower Grade Glioma | upregulate | Cell proliferation |  | [25] |
| Melanoma | upregulate | Cell proliferation, migration, invasion | miR-3182/NUPR1 | [5] |
| non-small cell lung cancer | upregulate | Cell proliferation, invasion, migration, EMT | miR-409-3p/HK2/LDHA, AKT/mTOR, miR-498, miR-26b-5p | [2, 22, 26-28] |
| Pancreatic Carcinoma | upregulate | Cell proliferation, migration, invasion | miR-448/WTAP/Fak, CDKN1A, KLF2 | [12, 32] |
| papillary thyroid carcinoma | upregulate | Cell proliferation, migration, invasion | miR-223-3p, SOS1， CCND1，c-myc | [16, 31] |
| oral cancer | upregulate | Cell proliferation | EZH2 | [4] |
| neuroblastoma | upregulate | Cell proliferation | miR-29, NOL4L | [15] |
| Osteosarcoma | upregulate | Cell proliferation | miR-635/TOP2A | [21] |

Table 3: Summary of potential targets and pathways of *DUXAP8* in cancers.
